# Detection of asymptomatic carotid stenosis in patients with lower-extremity arterial disease: development and external validations of a risk score

**DOI:** 10.1093/bjs/znab040

**Published:** 2021-04-19

**Authors:** M H F Poorthuis, D R Morris, G J de Borst, M L Bots, J P Greving, F L J Visseren, P Sherliker, R Clack, R Clarke, S Lewington, R Bulbulia, A Halliday

**Affiliations:** 1 Clinical Trial Service Unit and Epidemiological Studies Unit, Nuffield Department of Population Health, University of Oxford, Oxford, UK; 2 Department of Vascular Surgery, University Medical Center Utrecht, Utrecht, the Netherlands; 3 Medical Research Council Population Health Research Unit, Nuffield Department of Population Health, University of Oxford, Oxford, UK; 4 Julius Centre for Health Sciences and Primary Care, University Medical Center Utrecht, Utrecht University, Utrecht, the Netherlands; 5 Department of Vascular Medicine, University Medical Center Utrecht, Utrecht University, Utrecht, the Netherlands; 6 Nuffield Department of Surgical Sciences, John Radcliffe Hospital, University of Oxford, Oxford, UK; 7 UKM Medical Molecular Biology Institute (UMBI), Universiti Kebangsaan Malaysia, Kuala Lumpur, Malaysia

## Abstract

**Background:**

Recommendations for screening patients with lower-extremity arterial disease (LEAD) to detect asymptomatic carotid stenosis (ACS) are conflicting. Prediction models might identify patients at high risk of ACS, possibly allowing targeted screening to improve preventive therapy and compliance.

**Methods:**

A systematic search for prediction models for at least 50 per cent ACS in patients with LEAD was conducted. A prediction model in screened patients from the USA with an ankle : brachial pressure index of 0.9 or less was subsequently developed, and assessed for discrimination and calibration. External validation was performed in two independent cohorts, from the UK and the Netherlands.

**Results:**

After screening 4907 studies, no previously published prediction models were found. For development of a new model, data for 112 117 patients were used, of whom 6354 (5.7 per cent) had at least 50 per cent ACS and 2801 (2.5 per cent) had at least 70 per cent ACS. Age, sex, smoking status, history of hypercholesterolaemia, stroke/transient ischaemic attack, coronary heart disease and measured systolic BP were predictors of ACS. The model discrimination had an area under the receiver operating characteristic (AUROC) curve of 0.71 (95 per cent c.i. 0.71 to 0.72) for at least 50 per cent ACS and 0.73 (0.72 to 0.73) for at least 70 per cent ACS. Screening the 20 per cent of patients at greatest risk detected 12.4 per cent with at least 50 per cent ACS (number needed to screen (NNS) 8] and 5.8 per cent with at least 70 per cent ACS (NNS 17). This yielded 44.2 and 46.9 per cent of patients with at least 50 and 70 per cent ACS respectively. External validation showed reliable discrimination and adequate calibration.

**Conclusion:**

The present risk score can predict significant ACS in patients with LEAD. This approach may inform targeted screening of high-risk individuals to enhance the detection of ACS.

## Teaser

Our risk score, that is based on routinely collected clinical characteristics and simple physical measurements, can predict the presence of significant asymptomatic carotid stenosis in patients with lower-extremity arterial disease reliably. It can be used to inform targeted screening of high-risk participants in whom asymptomatic carotid stenosis is more often detected

## Introduction

Carotid stenosis is a cause of cerebral infarction in around 15 per cent of ischaemic strokes^1^. Significant asymptomatic carotid stenosis (ACS) is also a predictor of coronary events and vascular death^2^^,^^3^, and the risk of both stroke and heart attack can be reduced with appropriate medical therapy. Using duplex ultrasound imaging, the prevalence of moderate or severe (50 per cent or greater) and severe (70 per cent or greater) ACS in the general population has been estimated to be 2.0 and 0.5 per cent respectively^4^. The prevalence is higher in patients with lower-extremity arterial disease (LEAD)^5^^,^^6^, but guideline recommendations^7–10^ for screening for significant ACS in patients with LEAD vary. Arguments for screening include initiation or improvement of preventive therapies, but also more intensive follow-up to maintain compliance and reduce the risk of subsequent vascular events^11^^,^^12^. Arguments against screening include the low prevalence of significant ACS, even among patients with LEAD, and, although medical therapy is indicated in patients with ACS, considerable disagreement exists about the net clinical benefit of routine use of carotid interventions in patients with medically treated ACS.

Risk prediction models allow targeted screening among patients with LEAD at particularly high risk of significant ACS based on multiple risk factors. The aim of this study was to perform a systematic review for published risk prediction models, and further to develop and validate a new risk prediction model for the detection of ACS: the Detection of Asymptomatic Carotid Stenosis in patients with Lower-Extremity Arterial Disease (DACS-LEAD) risk score.

## Methods

A systematic review was performed to identify established risk prediction models for the prediction of at least 50 per cent ACS in patients with LEAD. This was conducted according to a predefined protocol that was registered prospectively in the international prospective registry for systematic reviews (PROSPERO) (number CRD42019155482) (*Appendix S1*).

### Derivation cohort

A risk prediction model was developed using data from 3 050 448 self-referred and self-funded individuals who attended commercial vascular screening clinics between 2008 and 2013 in the USA^13^^,^^14^. This was referred to as the derivation cohort. All individuals completed a standard questionnaire including questions about their age, sex, height and weight, smoking status, history of hypertension, hypercholesterolaemia, diabetes mellitus and vascular disease (transient ischaemic attack (TIA), stroke and coronary heart disease (CHD)), and use of antiplatelet, antihypertensive and lipid-lowering medication. BP was measured as part of the ankle : brachial pressure index (ABPI) assessment. Standard BP cuffs and sphygmomanometers were used, and systolic BP (SBP) was measured using a Doppler probe.

Most participants underwent carotid duplex screening (conducted by trained staff using dedicated vascular ultrasound instruments: LOGIQ e^®^; GE Healthcare). The highest peak systolic velocity (PSV) and end diastolic velocity (EDV) of both common and internal carotid arteries were measured.

LEAD was defined as an ABPI of 0.9 or less at either side, and these individuals were included in the present analyses. Participants were excluded if they did not undergo ABPI measurement, if ABPI determination was not possible because the arteries could not be compressed (175 517 patients), if ABPI was >0.9 on both sides (2 759 591), if they did not undergo duplex ultrasound imaging (2759) or of they had inconsistent values (464).

### External validation cohorts

The risk prediction model was validated by assessing the predictive performance in two independent (external) cohorts. For the first external validation of the risk prediction model, data for 225 691 self-referred and self-funded individuals who attended commercial vascular screening clinics between 2008 and 2013 in the UK were used. The following participants were excluded: those who did not undergo ABPI measurement or in whom ABI was not possible because the arteries could not be compressed (10 774 patients) or in whom ABPI was above 0.9 on both sides (209 276), those who did not undergo duplex ultrasonography (106), or who had inconsistent values (135).

For the second external validation, data from the Second Manifestation of ARTerial disease (SMART) study were used. This is an ongoing prospective cohort at the University Medical Centre Utrecht, the Netherlands. The rationale and design of the SMART study have been published previously^15^. Between September 1996 and October 2019, 13 799 patients with recent diagnosis (1 year before baseline) of a first manifestation of arterial disease, including cerebrovascular disease, coronary artery disease, peripheral arterial disease or aneurysm of the abdominal aorta, were included. After inclusion, individuals completed a questionnaire with questions about medical health and lifestyle, and underwent a standardized vascular screening. Office BP was measured with a non-random sphygmomanometer and the mean of multiple measurements was taken. Use of antithrombotic, BP lowering and cholesterol-lowering medication was recorded. For external validation, baseline characteristics of individuals who had ABPI determination and duplex ultrasound imaging of the carotid arteries were used. Patients with an ABPI above 0.9 on both sides (11 371 patients), in whom no duplex ultrasonography was performed (35), or who had recent (less than 12 months) cerebrovascular symptoms (220) were excluded.

### Predicted outcomes

Two outcomes were used. The first, moderate or severe ACS, was defined as at least 50 per cent ACS, based on a PSV of 150 cm/s or more on either side, or 0 cm/s for occluded arteries. The second outcome, severe ACS, was defined as at least 70 per cent ACS; based on a PSV of 210 cm/s or more on either side, or 0 cm/s for occluded arteries.

### Model derivation

The development of the prediction model adhered to the Transparent Reporting of a multivariable prediction model for Individual Prognosis Or Diagnosis (TRIPOD) statement^16^.

Baseline characteristics are presented as mean(s.d.) values for continuous variables and as absolute numbers and percentages for categorical variables. Age was categorized into four groups (<50, 50–59, 60–69 and ≥70 years), SBP into three groups (<140, 140–159 and ≥160 mmHg), ABPI into three groups (>0.8 to ≤0.9, more than >0.4 to ≤0.8, ≤0.4), and smoking status was dichotomized as ever smoking *versus* never smoking. For most predictors, the proportion of individuals with missing data was acceptable (less than 10 per cent), except for smoking status (10.5 per cent), reported history of CHD (13.8 per cent) and stroke/TIA (15.2 per cent) (*Table S1*). Missing data were multiple-imputed using chained equations, and 20 data sets with 200 iterations were created. Results were combined with Rubin’s rules^17^^,^^18^.

**Table 1:** Baseline characteristics of patients in the derivation and validation cohorts

	All patients	Patients with <50% ACS	Patients with 50–69% ACS	Patients with ≥70% ACS
**Derivation cohort: Life Line Screening (US patients)**	*n* = 112 117	*n* = 105 763	*n* = 3553	*n* = 2801^§^
Age (years)*	70.5(10.7)	70.3(10.8)	74.1(8.8)	72.4(9.1)
Male sex	31 004 (27.7)	28 505 (27.0)	1265 (35.6)	1234 (44.1)
Smoking status	*n*=100 376	*n*=94 717	*n*=3151	*n*=2508
Current smoker	19 706 (19.6)	18 089 (19.1)	814 (25.8)	803 (32.0)
Former smoker	38 851 (38.7)	36 231 (38.3)	1479 (46.9)	1141 (45.5)
Never smoked	41 819 (41.7)	40 397 (42.7)	858 (27.2)	564 (22.5)
Diabetes mellitus	21 549 of 102 019 (21.1)	19 951 of 96 218 (20.7)	951 of 3257 (29.2)	647 of 2544 (25.4)
Hypercholesterolaemia	54 271 of 105 607 (51.4)	50 671 of 99 662 (50.8)	2048 of 3325 (61.6)	1552 of 2620 (59.2)
CHD^†^	15 099 of 96 599 (15.6)	13 587 of 91 015 (14.9)	844 of 3144 (26.8)	668 of 2440 (27.4)
Stroke or TIA	9103 of 95 083 (9.6)	8217 of 89 639 (9.2)	446 of 3052 (14.6)	440 of 2392 (18.4)
SBP (mmHg)*	144.8(23.9)	144.4(23.7)	151.7(24.8)	152.3(25.6)
ABPI*^‡^	0.8 (0.1)	0.8 (0.1)	0.7 (0.2)	0.7 (0.2)
Aspirin	43 316 of 86 319 (50.2)	40 256 of 81 392 (49.5)	1715 of 2774 (61.8)	1345 of 2153 (62.5)
Lipid-lowering therapy	48 224 of 103 594 (46.6)	44 766 of 97 715 (45.8)	1958 of 3293 (59.5)	1500 of 2586 (58.0)
Antihypertensive therapy	65 323 of 104 546 (62.5)	60 855 of 98 590 (61.7)	2556 of 3355 (76.2)	1912 of 2601 (73.5)

**Validation cohort: Life Line Screening (UK patients)**	*n* = 5400	*n* = 4909	*n* = 230	*n* = 261^¶^
Age (years)*	70.3 (9.5)	70.0 (9.6)	73.2 (8.4)	73.2 (7.7)
Male sex	2007 (37.2)	1775 (36.2)	101 (43.9)	131 (50.2)
Smoking status	*n*=4643	*n*=4224	*n*=202	*n*=217
Current smoker	1497 (32.2)	1333 (31.6)	79 (39.1)	85 (39.2)
Former smoker	1675 (36.1)	1509 (35.7)	79 (39.1)	87 (40.1)
Never smoked	1471 (31.7)	1382 (32.7)	44 (21.8)	45 (20.7)
Diabetes mellitus	711 of 4262 (16.7)	637 of 3890 (16.4)	34 of 183 (18.6)	40 of 189 (21.2)
Hypercholesterolaemia	1899 of 4237 (44.8)	1696 of 3885 (43.7)	92 of 174 (52.9)	111 of 178 (62.4)
CHD^†^	655 of 4178 (15.7)	559 of 3818 (14.6)	37 of 179 (20.7)	59 of 181 (32.6)
Stroke or TIA	377 of 4075 (9.3)	321 of 3738 (8.6)	24 of 167 (14.4)	32 of 170 (18.8)
SBP (mmHg)*	149.8 (24.6)	149.0 (24.4)	155.7 (24.0)	158.9 (25.6)
ABPI*^‡^	0.8 (0.1)	0.8 (0.1)	0.7 (0.1)	0.7 (0.2)
Aspirin	952 of 2479 (38.4)	832 of 2256 (36.9)	51 of 98 (52.0)	69 of 125 (55.2)
Lipid-lowering therapy	1849 of 4220 (43.8)	1636 of 3873 (42.2)	102 of 172 (59.3)	111 of 175 (63.4)
Antihypertensive therapy	2205 of 4297 (51.3)	1963 of 3936 (49.9)	116 of 179 (64.8)	126 of 182 (69.2)

**Validation cohort: SMART**	*n* = 1536	*n* = 1278	*n* = 67	*n* = 191^#^
Age (years)*	61.6 (10.3)	60.7 (10.5)	65.7 (6.9)	65.8 (7.9)
Male sex	1021 (66.5)	838 (65.6)	45 (67.2)	138 (72.3)
Smoking status	*n*=1522	*n*=1266	*n*=67	*n*=189
Current smoker	775 (50.9)	648 (51.2)	33 (49.3)	94 (49.7)
Former smoker	601 (39.5)	490 (38.7)	25 (37.3)	86 (45.5)
Never smoked	146 (9.6)	128 (10.1)	9 (13.4)	9 (4.8)
Diabetes mellitus	354 of 1536 (23.0)	279 of 1278 (21.8)	19 of 67 (28.4)	56 of 191 (29.3)
Hypercholesterolaemia	852 of 1499 (56.8)	706 of 1248 (56.6)	35 of 65 (53.8)	111 of 186 (59.7)
CHD^†^	339 of 1534 (22.1)	264 of 1276 (20.7)	20 of 67 (29.9)	55 of 191 (28.8)
Stroke or TIA	110 of 1532 (7.2)	71 of 1274 (5.6)	4 of 67 (6)	35 of 191 (18.3)
SBP (mmHg)*	147 (22.9)	146 (22.8)	148 (23.6)	153 (22.8)
ABPI*^‡^	0.7 (0.2)	0.7 (0.2)	0.6 (0.2)	0.6 (0.2)
Antiplatelet therapy	721 of 1356 (53.2)	617 of 1150 (53.7)	26 of 56 (46.4)	78 of 150 (52)
Anticoagulant	136 of 1483 (9.2)	112 of 1244 (9)	5 of 64 (7.8)	19 of 175 (10.9)
Lipid-lowering therapy	866 of 1536 (56.4)	700 of 1278 (54.8)	40 of 67 (59.7)	126 of 191 (66)
Antihypertensive therapy	993 of 1536 (64.6)	799 of 1278 (62.5)	50 of 67 (74.6)	144 of 191 (75.4)

Values in parentheses are percentages unless indicated otherwise; *values are mean(s.d.). ^†^Coronary heart disease (CHD) is defined as previous myocardial infarction or coronary intervention (bypass, angioplasty or stenting). ^‡^The lowest ankle : brachial pressure index (ABPI) value of both lower extremities was included. ^§^In this group, 629 patients in the derivation cohort had a presumed occlusion. ^¶^In this group, 41 patients in the validation cohort had a presumed occlusion. ^#^In this group, 67 patients in the validation cohort had a presumed occlusion. ACS, asymptomatic carotid stenosis; TIA, transient ischaemic attack; SBP, systolic BP; SMART, Second Manifestation of ARTerial disease.

The relationship between predictors and the presence of at least 50 per cent and at least 70 per cent ACS in individuals with LEAD was determined with multivariable logistic regression. Predictors were selected based on risk prediction models for significant ACS in the general population^19^, and forward stepwise selection with predictors selected using Akaike information criterion (AIC)^20^. Discrimination and calibration of the developed risk prediction model were examined. Discrimination is the ability of the prediction model to distinguish between patients with and those without the disease outcomes, assessed using the area under the receiver operating characteristic (AUROC) curve. Calibration is the agreement between predicted and observed risk, and was assessed with calibration plots.

### Internal validation, score chart and targeted screening

Internal validation was performed to control for potential overfitting. Overfitting quantifies the difference in predictive performance between the data from which the risk prediction model is derived and similar future cases. The predictive performance is expected to be better in the derivation cohort because the logistic model fits the data in an optimal fashion. Bootstrapping to sample the original data set was used and 1000 bootstrap replications per imputed data set were created^21^. The mean calibration slope of the 1000 bootstrap replications in each imputed data set was calculated and used as a uniform shrinkage factor to adjust the regression coefficients for risk of potential overfitting. The shrunken *β* coefficients were used to calculate the adjusted intercept by fitting a logistic model with the shrunken *β* coefficients as dependent variables in the original data set. Overoptimism-corrected AUROC curves for each imputed data set were calculated, and the results were combined with Rubin’s rules^17^^,^^18^.

Regression coefficients of the predictors were converted into points on a score chart to facilitate clinical use of the risk prediction model. One score chart was created for ACS of both 50 and 70 per cent or above. For this, the *β* coefficients were multiplied by four and then rounded to the closed integer. If the scores for at least 50 and 70 per cent ACS were conflicting, the score for at least 50 per cent ACS was used. The risk of ACS of at least 50 and 70 per cent for the total points (sum scores) was calculated.

Test characteristics of a targeted screening programme using this risk score for selection of participants were calculated. The number needed to screen (NNS) to detect participants with ACS, positive and negative predictive values, sensitivity and specificity for four possible thresholds for targeted screening were calculated.

### External validation

External validation assesses the predictive performance of the developed risk prediction model in independent cohorts. Participants who attended commercial vascular screening clinics in the UK and patients from the SMART cohort were used for external validation^15^. The same methods for reporting baseline characteristics and handling missing data were used as in the derivation cohort. The original regression formula (after internal validation) was used to calculate the risk of at least 50 and 70 per cent ACS. Discrimination was assessed using the AUROC curve, and calibration with calibration plots. All predictors could be matched with variables in the validation cohorts, but a proxy was used for history of hypercholesterolaemia in one external validation cohort. The screening approach was based on self-report in the derivation cohort and on blood measurement in the SMART cohort.

Differences between the prevalence of the predicted outcome in the development cohort and the validation cohorts are known to influence calibration. For this reason, the DACS-LEAD was recalibrated to the prevalence of the predicted outcome in the external validation cohorts by adjusting the original intercept. This type of recalibration is referred to as ‘update intercept’ or ‘calibration-in-the-large’^22^. STATA^®^ version 15.1 (StataCorp, College Station, TX, USA) was used for all statistical analyses and R version 3.5.1 (The R Foundation for Statistical Computing, Vienna, Austria) for constructing figures.

## Results

Some 4907 unique reports identified by literature searching were screened and 43 full-text reports were assessed for eligibility. No study met the inclusion criteria (*Fig. S1* and *Table S2*), and no external validation of established risk prediction models could be performed.

**Fig. 1:**
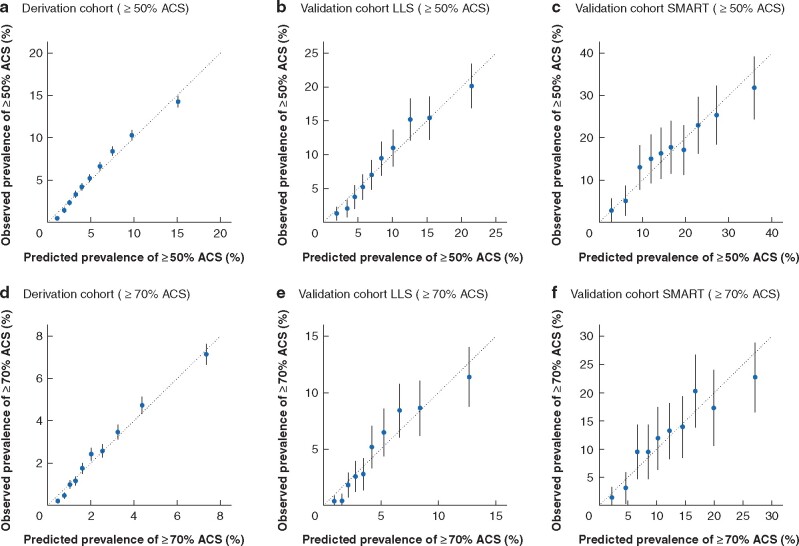
Calibration plots of Detection of Asymptomatic Carotid Stenosis in patients with Lower-Extremity Arterial Disease (DACS-LEAD) in derivation and validation cohorts Plots show predicted *versus* observed risk of at least 50 and 70 per cent asymptomatic carotid stenosis across deciles of predicted risk in **a,d** the derivation cohort after internal validation, **b,e** the Life Line Screening (LLS) (UK) validation cohort, and **c,f** the SMART (Second Manifestation of ARTerial disease) cohort after recalibration. The mean (95 per cent c.i.) predicted risk for each decile is shown. The diagonal line indicates perfect calibration. Values above the diagonal line indicate underestimation of risk and those below the diagonal line indicate overestimation of risk. Calibration plots of DACS-LEAD in the validation cohorts before recalibration are shown in *Fig. S3*. Prevalences and number of cases per decile are given in *Table S3*.

**Table 2:** Predictors of moderate and severe asymptomatic carotid stenosis in patients with lower-extremity arterial disease

	Odds ratio*	
	≥50% ACS	≥70% ACS
**Predictors^‡^**		
Age (years) (reference <50 years)		
50–59	3.27 (2.11, 5.09)	3.26 (1.78, 5.99)
60–69	5.15 (3.34, 7.95)	4.41 (2.42, 8.01)
≥70	5.76 (3.74, 8.87)	4.16 (2.29, 7.55)
Male sex	1.35 (1.27, 1.42)	1.60 (1.48, 1.73)
Ever smoking	1.94 (1.82, 2.07)	2.07 (1.88, 2.28)
Hypercholesterolaemia	1.35 (1.28, 1.42)	1.27 (1.17, 1.38)
Stroke or TIA	1.42 (1.31, 1.54)	1.63 (1.46, 1.82)
CHD	1.52 (1.42, 1.62)	1.44 (1.31, 1.58)
SBP (mmHg) (reference <140 mmHg)		
140–159	1.24 (1.16, 1.32)	1.26 (1.14, 1.38)
≥160	1.73 (1.63, 1.85)	1.80 (1.63, 1.98)
ABPI (reference >0.8–≤0.9)		
>0.4–≤0.8	2.08 (1.97, 2.20)	2.15 (1.98, 2.34)
≤0.4	3.62 (3.18, 4.12)	3.69 (3.08, 4.43)
Intercept^§^	−5.93	−6.69
**Discrimination derivation cohort^¶^**		
After internal validation	0.71 (0.71, 0.72)^†^	0.73 (0.72, 0.73)^†^
**Discrimination validation cohorts**		
In Life Line Screening UK patients	0.70 (0.68, 0.73)^†^	0.72 (0.69, 0.74)^†^
In SMART study	0.67 (0.63, 0.70)^†^	0.67 (0.64, 0.70)^†^

*With 95 per cent c.i. in parentheses except where indicated otherwise; ^†^AUROC curve values with 95 per cent confidence intervals. The original regression formula can be derived from the odds ratios and the intercept. The *β* coefficients for the linear predictor (LP) can be calculated by taking the natural logarithm of the odds ratios. The LP can be calculated with the following formula: LP = Intercept + *β*_1_*x*_1_ + *β*_2_*x*_2_ + *β*_3_*x*_3_ … *β_n_x_n_*, where the *β*s are the *β* coefficients or weights of the predictors and the *x*s are the predictors. The predicted probability can be calculated by: *e*^LP^/(1 + *e*^LP^). ^‡^*β* coefficients and intercept corrected for overoptimism with bootstrapping techniques (shrinkage of regression coefficients was not indicated with calibration slope of 1.00). ^§^Bootstrap-adjusted intercepts were the same as the intercept before internal validation. ^¶^Area under the receiver operating characteristic (AUROC) curves before internal validation was 0.71 (95 per cent c.i. 0.71 to 0.72) for at least 50 per cent asymptomatic carotid stenosis (ACS) and 0.73 (0.72 to 0.73) for at least 70 per cent ACS. TIA, transient ischaemic attack; CHD, coronary heart disease; SBP, systolic BP; ABPI, ankle : brachial pressure index; SMART, Second Manifestation of ARTerial disease.

### Derivation cohort

In total, 112 117 patients with LEAD were used for development. The mean(s.d.) age in the derivation cohort was 70.5(10.7) years and 27.7 per cent were men. Around 50 per cent of patients reported use of aspirin and lipid-lowering therapy, and about 60 per cent reported use of antihypertensive therapy. In patients with significant ACS, around 60 per cent reported use of aspirin and lipid-lowering therapy, and almost 75 per cent reported use of antihypertensive therapy. The overall prevalence of at least 50 per cent ACS in patients with LEAD was 5.7 per cent and that for at least 70 per cent ACS was 2.5 per cent (*Table 1*).

### Risk prediction model development and internal validation

The following predictors were included: age, sex, ever smoking versus never smoking, history of hypercholesterolaemia, stroke/TIA, CHD, measured SBP, and ABPI. The AUROC curve adjusted for overoptimism was 0.71 (95 per cent c.i. 0.71 to 0.72) for at least 50 per cent ACS and 0.73 (0.72 to 0.73) for at least 70 per cent ACS (*Table 2*). Internal validation with bootstrapping techniques indicated that no shrinkage of the *β* coefficients was needed. Calibration plots showed good concordance between predicted and observed risk for ACS of both at least 50 and 70 per cent, indicating that groups of patients at both low and high risk can be predicted reliably by the DACS-LEAD risk score (*Fig. 1a,d*).

### Targeted screening to detect asymptomatic carotid stenosis

**Fig. 2:**
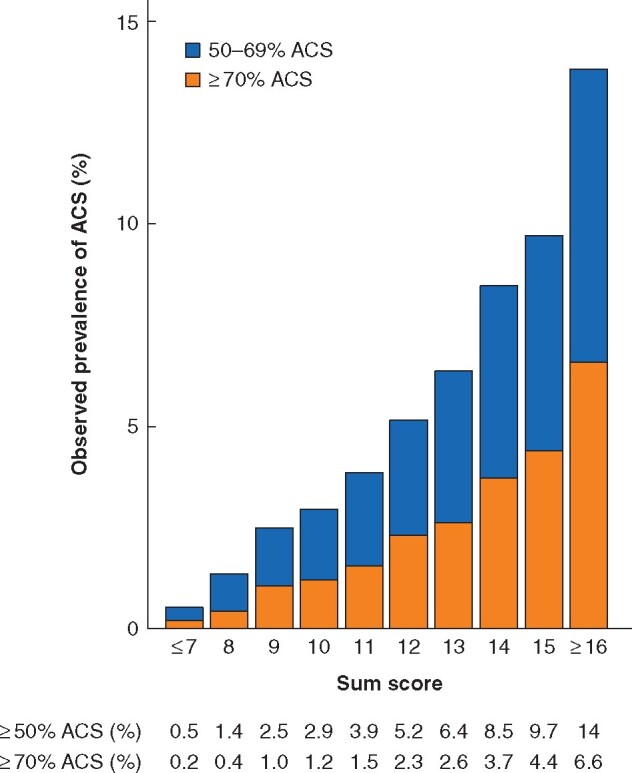
Bar chart showing the predicted prevalence of asymptomatic carotid stenosis in the derivation cohort for each sum score ACS, asymptomatic carotid stenosis.

The DACS-LEAD score chart is provided in *Table 3*. Sum scores ranged from 0 to 22. The prevalence of at least 50 per cent ACS ranged from 0.5 per cent for sum scores of 7 or less to 14 per cent for sum scores of 16 or more. The prevalence of at least 70 per cent ACS ranged from 0.2 per cent for sum scores of 7 or less to 6.6 per cent for sum scores of 16 or more. The prevalence of at least 50 and 70 per cent ACS by each sum score is shown in *Fig. 2. *The distribution of sum scores is shown in *Fig. S2*.

**Table 3:** Detection of Asymptomatic Carotid Stenosis in patients with Lower-Extremity Arterial Disease (DACS-LEAD) **score chart**

Predictor	Score
**Age (years)**	
<50	0
50–59	5
≥60	7
**Male sex**	1
**Ever smoking**	3
**Hypercholesterolaemia**	1
**Stroke or TIA**	1
**CHD**	2
**SBP (mmHg)**	
<140	0
140–159	1
≥160	2
**ABPI**	
>0.8–≤0.9	0
>0.4–≤0.8	3
≤0.4	5

The DACS-LEAD score ranged from 0 to 22. Risks of at least 50 and 70 per cent asymptomatic carotid stenosis for each sum score are shown in *Fig. 2*. TIA, transient ischaemic attack; CHD. coronary heart disease; SBP, systolic BP; ABPI, ankle : brachial pressure index.

**Table 4:** Performance of the risk scores to detect asymptomatic carotid stenosis

	No. of patients screened	No. with ACS	NNS	PPV (observed prevalence) (%)	NPV (%)	Sensitivity (%)	Specificity (%)
**Systematic screening of all patients**							
≥50% ACS	112 117	6354	18	5.7			
≥70% ACS	112 117	2801	40	2.5			
**Screening of those with at least modest risk (sum score ≥9)**							
≥50% ACS	92 630	6193	15	6.7	99.2	97.5	18.3
≥70% ACS	92 630	2747	33	3.0	99.7	98.1	17.8
**Screening of those with at least intermediate risk (sum score ≥11)**							
≥50% ACS	71 521	5639	13	7.9	98.2	88.8	37.7
≥70% ACS	71 521	2521	29	3.5	99.3	90.0	36.9
**Screening of those with at least high risk (sum score ≥13)**							
≥50% ACS	44 713	4435	10	9.9	97.2	69.8	61.9
≥70% ACS	44 713	2010	22	4.5	98.8	71.8	60.9
**Screening of those with very high risk (sum score ≥15)**							
≥50% ACS	22 704	2809	8	12.4	96.0	44.2	81.2
≥70% ACS	22 704	1313	17	5.8	98.3	46.9	80.4

The number of false negatives and true negatives was 161 and 19 326, 715 and 39 881, 1919 and 65 485, and 3545 and 85 868 for sum scores for at least 50 per cent asymptomatic carotid stenosis (ACS) of 9 or more, 11 or more, 13 or more, and 15 or more respectively, and 54 and 19 433, 280 and 40 316, 791 and 66 613, and 1488 and 87 925 for respective sum scores for at least 70 per cent ACS. NNS, number needed to screen; PPV, positive predictive value; NPV, negative predictive value.

Four thresholds of sum scores were introduced, allowing targeted screening of a group of patients with LEAD at high risk of significant ACS. The observed prevalence of at least 50 per cent ACS increased from 5.7 per cent by screening all patients to 12.4 per cent by targeted screening of the 20 per cent of patients at very high risk. The corresponding NNS decreased from 18 to 8. The observed prevalence of at least 70 per cent ACS increased from 2.5 per cent by screening all patients to 5.8 per cent by targeted screening of the 20 per cent of patients at very high risk. The corresponding NNS decreased from 40 to 17 (*Table 4*).

### External validation populations

In the first validation cohort (Life Line Screening; UK participants), compared to the derivation cohort more patients were men (37.2 per cent) and current smokers (32.2 per cent), and fewer patients had diabetes mellitus (16.7 per cent) and hypercholesterolaemia (44.8 per cent) (*Table 1*). The prevalence of at least 50 per cent ACS was 9.1 per cent and that for at least 70 per cent ACS was 4.8 per cent. In the second validation cohort (SMART), patients were younger (mean(s.d.) age 61.6(10.3) years) and more were men (66.5 per cent) than in the derivation cohort. The prevalence of at least 50 per cent ACS was 16.8 per cent and that for at least 70 per cent ACS was 12.4 per cent.

### External validation

The AUROC of DACS-LEAD in the first external validation cohort was 0.70 (95 per cent c.i. 0.68 to 0.73) for at least 50 per cent ACS and 0.72 (0.69 to 0.74) for at least 70 per cent ACS. Respective values in the second validation cohort were 0.67 (0.63 to 0.70) and 0.67 (0.64 to 0.70) (*Table 2*). Risks were underestimated when the risk score was tested in the validation cohorts (*Fig. S3*), but calibration plots showed good concordance between the predicted prevalence calculated with the DACS-LEAD and the observed prevalence in the validation cohorts (after adjusting the intercept) (*Fig. 1*). This indicates that, after adjusting the mean predicted risk to the observed risk in the validation cohorts, the DACS-LEAD risk score could be applied to populations with LEAD with different overall prevalence of significant ACS.

## Discussion

Predictors for moderate and severe ACS used in the DACS-LEAD risk score were age, sex, ever smoking, history of hypercholesterolaemia, stroke/TIA, CHD, and measured SBP and ABPI. Discrimination was good, and calibration plots showed good concordance of predicted and observed risks. The NNS to detect significant ACS in the high-risk group that consisted of approximately 20 per cent patients at highest risk was more than halved compared with systematic screening of all patients with LEAD. Application of DACS-LEAD to the validation cohort showed reliable predictions (after adjusting for the difference in overall prevalence of significant ACS in the different validation cohorts).

The DACS-LEAD risk score can be applied to patients with decreased ABPI of 0.9 or below. These ABPI values have been associated with cardiovascular morbidity and mortality, and might also improve cardiovascular risk stratification^23^^,^^24^. The prevalence of significant ACS is higher in patients with LEAD and increases with the severity of LEAD^25^. A meta-analysis^6^ including 13 prospective studies of patients with LEAD showed a prevalence of at least 50 per cent ACS of 25 per cent, and a prevalence of at least 70 per cent ACS of 14 per cent, but heterogeneity between studies was high owing to different selection criteria and the application of different diagnostic criteria to determine the degree of carotid stenosis. The prevalence in the validation cohorts was higher than in the derivation cohort due to the inclusion of patients with a first manifestation of arterial disease in the SMART cohort, and possibly different reasons for undergoing vascular screening in the USA and the UK. This hampers direct implementation of the present risk score in clinical practice. Adjustment of the prediction model (while keeping the included predictors) to new settings is necessary. Another approach might be to develop different risk models based on the disease severity of LEAD. The Fontaine or Rutherford classifications could be used for patient selection, and the risks of ACS calibrated accordingly (for example, separate predictions for Fontaine stage IIb–IV *versus* I–IIa) to provide more consistent predictions of ACS across cohorts.

Detection of significant ACS in patients with asymptomatic LEAD might lead to the initiation or intensification of medical therapy. In patients with symptomatic LEAD who were receiving optimal medical therapy, detection might require more intensive follow-up to maintain compliance^11^^,^^12^. Compliance with medical therapy is challenging, as recently shown in a study from Sweden^25^. In this study of screening for ACS in men aged 65 years, statins and antiplatelet agents were prescribed when ACS was detected, but were used by 29 and 21 per cent of patients respectively at 5 years after detection, compared with approximately 23 and 14 who used them at age 65 years^26^. Furthermore, intensification of both lipid-lowering therapy^27^^,^^28^, antiplatelet therapy^29^, and a combination of low-dose aspirin and low-dose rivaroxaban might be particularly beneficial in terms of stroke prevention in patients with significant ACS^30^.

Although the annual risk of ipsilateral stroke in patients with significant ACS using best medical therapy is low^31^^,^^32^, the 5–10-year risk may not be negligible, especially in patients with severe ACS.^33^ Risk factors for increased stroke risk in medically treated patients have been identified, but risk models have not been validated in patients using current evidence-based medical therapy^34^^,^^35^. Carotid interventions might be considered in selected patients to reduce stroke risk further, but the benefit of carotid interventions should, amongst other considerations, be weighed against the limited life expectancy in patients with LEAD^9^.

Participants of the derivation cohort and one of the validation cohorts were self-referred and self-funded, possibly influencing generalizability to other populations. The derivation cohort was not designed primarily for research purposes, but participants were identified prospectively. ABPI and PSV as single measurements for diagnosing LEAD and ACS may be useful as a screening tool to identify patients for more intensive diagnostic work-up. Substantial differences in the overall prevalence of ACS across cohorts were found. The timing of stroke/TIA was not recorded, and some patients may have had recent cerebrovascular symptoms from their carotid stenosis. For predictors that were self-reported, recall bias should be taken into account. BP was measured once in the US and UK cohorts, and might not reflect ‘usual’ values. A proxy for history of hypercholesterolaemia was used in the SMART cohort, which might have influenced external validity. The prevalence of ACS in the derivation cohort was lower than in other populations, possibly making targeted screening more worthwhile in different settings^6^.

The present study has several strengths. The first risk score to detect significant ACS in individuals with LEAD was developed and validated in two populations. A large cohort of individuals was used for development of the DACS-LEAD risk score. Missing data were limited for most predictors in the derivation and validation cohorts, and multiple imputation was used to handle missing data. Internal validation showed no evidence of overfitting, and external validation showed reliable prediction after recalibration. ABPI was measured bilaterally, and individuals with incompressible ankle arteries or an ABPI above 1.4 were excluded. Individuals underwent bilateral examination of the carotid arteries, and the highest degree of stenosis of both sides was used as outcome.

Before targeted screening can be implemented in clinical practice, future research will determine risks of cardiovascular events in individuals with LEAD and concomitant significant ACS under best medical therapy, will identify patients with significant ACS at increased risk of ischaemic stroke who benefit from carotid interventions, and whether closer follow-up and better compliance after detecting significant ACS might improve cardiovascular risk management and prevent cardiovascular events.

The DACS-LEAD risk score can provide individualized predictions of the presence of moderate and severe ACS in individuals with LEAD, using the following predictors: age, sex, smoking status, history of hypercholesterolaemia, stroke/TIA, CHD, and measured SBP. Using the risk score to select individuals for a targeted screening programme, approximately 40 per cent of patients with ACS were detected by screening only 20 per cent of individual participants. The yield increased to approximately 70 per cent when 40 per cent of participants were screened. The NNS was reduced substantially, and the present risk score could help to target screening in those whom ACS is more likely to be detected.

## Supplementary Material

znab040_Supplementary_Appendix
