# Supplementary material for: Detection of asymptomatic carotid stenosis in patients with lower-extremity arterial disease: development and external validations of a risk score
Source: Br J Surg. 2021 Apr 19;108(8):960–7. doi: 10.1093/bjs/znab040 (PMC10364916; doi:10.1093/bjs/znab040)
Supplement: znab040_Supplementary_Appendix [file znab040_supplementary_appendix.docx]

# Detection of Asymptomatic Carotid Stenosis in Patients with Lower Extremity Arterial Disease: development and external validations of a risk score

**Supplement**

# **Table** **of** **contents**

[Appendix Table 1. Search strategy 3](#_Toc57627737)

[Appendix Table 2. TRIPOD Checklist 5](#_Toc57627741)

[Appendix Table 3. Missing data per variable 7](#_Toc57627742)

[Appendix Table 4. Full-text evaluation 8](#_Toc57627743)

[Appendix Table 5. Predicted and observed prevalence of ACS across deciles of predicted risk 9](#_Toc57627744)

[Appendix Figure 1. Flowchart 10](#_Toc57627745)

[Appendix Figure 2. Distribution of sum scores in the derivation cohort 11](#_Toc57627746)

[Appendix Figure 3. Calibration plots of DACS-LEAD in validation cohort (before recalibration) 12](#_Toc57627747)

[References 13](#_Toc57627748)

**Appendix Table 1. Search strategy**

| **Medline (via PubMed interface)**  1. "Carotid Stenosis"[Mesh]  2. "Carotid stenosis"[tiab] OR "Carotid artery stenosis"[tiab] OR "Carotid artery occlusion"[tiab] OR "Carotid artery stenoses"[tiab]  3. #1 OR #2  4. (Validat$ OR Predict$.ti. OR Rule$) OR (Predict$ AND (Outcome$ OR Risk$ OR Model$)) OR ((History OR Variable$ OR Criteria OR Scor$ OR Scoring$ OR Characteristic$ OR Finding$ OR Factor$) AND (Predict$ OR System$ OR Model$ OR Decision$ OR Identif$ OR Prognos$)) OR (Decision$ AND (Model$ OR Clinical$ OR Logistic Models/)) OR (Prognostic AND (History OR Variable$ OR Criteria OR Scor$ OR Characteristic$ OR Finding$ OR Factor$ OR Model$))^1^  5. "Mass Screening"[Mesh] OR Screen*[tiab]  6. Prevalence[Mesh] OR prevalenc* OR communit*[tiab]  7. "Population"[MeSH] OR population*[tiab]  8. "Peripheral Arterial Disease"[MeSH]  9. "Peripheral arterial disease"[tiab] OR "Limb ischaemia "[tiab] OR "Limb ischemia "[tiab]  10. "Aortic aneurysm"[MeSH Terms] OR "aortic aneurysm, abdominal"[MeSH Terms]  11. "Aortic aneurysm"[tiab]  12. #5 OR #6 OR #7 OR #8 OR #9 OR #10 OR #11  13. #3 AND #4 AND #12  ------------  324 references identified on December 1, 2019 |
| --- |

| **EMBASE (via OVID EMBASE interface) ^^[[1]](#footnote-1)^^**  1. exp carotid artery stenosis/  2. (carotid artery or carotid artery atherosclerosis or carotid artery disease or carotid artery diseases).ti,ab,kw.  3. stenos*.ti,ab,tw.  4. 2 and 3  5. 1 or 4  6. predict.ti.  7. (validat* or rule*).ti,ab.  8. (predict* and (outcome* or risk* or model*)).ti,ab.  9. ((history or variable* or criteria or scor* or characteristic* or finding* or factor*) and (predict* or model* or decision* or identif* or prognos*)).ti,ab.  10. decision*.ti,ab. and statistical model/  11. (decision* and (model* or clinical*)).ti,ab.  12. (prognostic and (history or variable* or criteria or scor* or characteristic* or finding* or factor* or model*)).ti,ab.  13. (stratification or discrimination or discriminate or c statistic or "area under the curve" or auc or calibration or indices or algorithm or multivariable).ti,ab.  14. receiver operating characteristic/  15. 6 or 7 or 8 or 9 or 10 or 11 or 12 or 13 or 14  16. exp mass screening/  17. Screening.ab,ti,kw.  18. exp prevalence/  19. Prevalence.ab,ti,kw.  20. exp peripheral occlusive artery disease/  21. (peripheral arterial disease or Limb ischaemia or Limb ischemia).ti,ab  22. exp abdominal aorta aneurysm/ or exp aortic aneurysm/ or exp aorta aneurysm/  23. (Aortic aneurysm).ti,ab  24. 16 or 17 or 18 or 19 or 20 or 21 or 22 or 23  25. 5 and 15 and 24  26. letter.pt. or letter/  27. note.pt.  28. conference abstract.pt.  29. editorial.pt.  30. case report/ or case study/  31. (letter or comment*).ti.  32. 26 or 27 or 28 or 29 or 30 or 31  33. animal/ not human/  34. nonhuman/  35. exp animal experiment/  36. exp experimental animal/  37. animal model/  38. exp rodent/  39. (rat or rats or mouse or mice).ti.  40. 33 or 34 or 35 or 36 or 37 or 38 or 39  41. 32 or 40  42. 25 not 41  43. limit 42 to embase  -------  4758 references identified on December 1, 2019 |
| --- |

**Appendix Table 2. TRIPOD Checklist**

| **Section/Topic** | **Item** | **Checklist Item** | **Page** |
| --- | --- | --- | --- |
| **Title and abstract** | | | |
| Title | 1 | Identify the study as developing and/or validating a multivariable prediction model, the target population, and the outcome to be predicted. | ✓ Title |
| Abstract | 2 | Provide a summary of objectives, study design, setting, participants, sample size, predictors, outcome, statistical analysis, results, and conclusions. | ✓ Abstract |
| **Introduction** | | | |
| Background and objectives | 3a | Explain the medical context (including whether diagnostic or prognostic) and rationale for developing or validating the multivariable prediction model, including references to existing models. | ✓ Intro |
|  | 3b | Specify the objectives, including whether the study describes the development or validation of the model or both. | ✓ Intro |
| **Methods** | | | |
| Source of data | 4a | Describe the study design or source of data (e.g., randomized trial, cohort, or registry data), separately for the development and validation data sets, if applicable. | ✓ Methods |
|  | 4b | Specify the key study dates, including start of accrual; end of accrual; and, if applicable, end of follow-up. | ✓ Methods |
| Participants | 5a | Specify key elements of the study setting (e.g., primary care, secondary care, general population) including number and location of centres. | ✓ Methods |
|  | 5b | Describe eligibility criteria for participants. | ✓ Methods |
|  | 5c | Give details of treatments received, if relevant. | NA |
| Outcome | 6a | Clearly define the outcome that is predicted by the prediction model, including how and when assessed. | ✓ Methods |
|  | 6b | Report any actions to blind assessment of the outcome to be predicted. | NA |
| Predictors | 7a | Clearly define all predictors used in developing or validating the multivariable prediction model, including how and when they were measured. | ✓ Methods |
|  | 7b | Report any actions to blind assessment of predictors for the outcome and other predictors. | NA |
| Sample size | 8 | Explain how the study size was arrived at. | ✓ Methods |
| Missing data | 9 | Describe how missing data were handled (e.g., complete-case analysis, single imputation, multiple imputation) with details of any imputation method. | ✓ Methods |
| Statistical analysis methods | 10a | Describe how predictors were handled in the analyses. | ✓ Methods |
|  | 10b | Specify type of model, all model-building procedures (including any predictor selection), and method for internal validation. | ✓ Methods |
|  | 10d | Specify all measures used to assess model performance and, if relevant, to compare multiple models. | ✓ Methods |
| Risk groups | 11 | Provide details on how risk groups were created, if done. | ✓ Methods |
| **Results** | | | |
| Participants | 13a | Describe the flow of participants through the study, including the number of participants with and without the outcome and, if applicable, a summary of the follow-up time. A diagram may be helpful. | ✓ Methods |
|  | 13b | Describe the characteristics of the participants (basic demographics, clinical features, available predictors), including the number of participants with missing data for predictors and outcome. | ✓ Results |
| Model development | 14a | Specify the number of participants and outcome events in each analysis. | ✓ Results |
|  | 14b | If done, report the unadjusted association between each candidate predictor and outcome. | NA |
| Model specification | 15a | Present the full prediction model to allow predictions for individuals (i.e., all regression coefficients, and model intercept or baseline survival at a given time point). | ✓ Table 2 |
|  | 15b | Explain how to the use the prediction model. | ✓ Results |
| Model performance | 16 | Report performance measures (with CIs) for the prediction model. | ✓ Results |
| **Discussion** | | | |
| Limitations | 18 | Discuss any limitations of the study (such as nonrepresentative sample, few events per predictor, missing data). | ✓ Discussion |
| Interpretation | 19b | Give an overall interpretation of the results, considering objectives, limitations, and results from similar studies, and other relevant evidence. | ✓ Discussion |
| Implications | 20 | Discuss the potential clinical use of the model and implications for future research. | ✓ Discussion |
| **Other information** | | | |
| Supplementary information | 21 | Provide information about the availability of supplementary resources, such as study protocol, Web calculator, and data sets. | ✓ Appendix |
| Funding | 22 | Give the source of funding and the role of the funders for the present study. | ✓ |

**Appendix Table 3.** **Missing data per variable**

| **Variable** | **Percentage of participants with missing** |
| --- | --- |
| ***Derivation cohort: Life Line Screening – US patients*** | |
| Age | 0 |
| Sex | 0 |
| Smoking status | 10.5 |
| Diabetes mellitus | 9.0 |
| Hypercholesterolemia | 5.8 |
| CHD | 13.8 |
| Stroke/TIA | 15.2 |
| SBP | 0.8 |
| ***Validation cohort: Life Line Screening – UK patients*** | |
| Age | 0 |
| Sex | 0 |
| Smoking status | 14.0 |
| Diabetes mellitus | 21.1 |
| Hypercholesterolemia | 21.5 |
| CHD | 22.6 |
| Stroke/TIA | 24.5 |
| SBP | 0.7 |
| ***Validation cohort: SMART study*** | |
| Age | 0 |
| Sex | 0 |
| Smoking status | 0.9 |
| Hypercholesterolemia | 2.4 |
| Diabetes mellitus | 0 |
| CHD | 0.1 |
| Stroke/TIA | 0.3 |
| SBP | 0.3 |
| CHD, coronary heart disease; SBP, systolic blood pressure; SMART, Second Manifestations of ARTerial disease; TIA, transient ischemic attack. | |

**Appendix Table 4.** **Full-text evaluation**

|  | **Reason for exclusion** | **Number of studies** |
| --- | --- | --- |
|  | Not population of interest | **9** ^2-10^ |
|  | Determination of risk factors of ACS without prediction model | **4** ^11-14^ |
|  | Prevalence of ACS estimated only | **6** ^15-20^ |
|  | Etiologic research determining risk factors for ACS | **7** ^21-27^ |
|  | Health-economic research on ACS | **2** ^28, 29^ |
|  | Outcome: progression/regression of ACS | **1** ^30^ |
|  | Review on screening | **4** ^31-34^ |
|  | Diagnostic research with other determinant and/or outcome | **3** ^35-37^ |
|  | Estimation of stroke risk or mortality in patients with ACS | **7** ^38-44^ |
| ACS, asymptomatic carotid stenosis. | | |

**Appendix Table 5. Predicted and observed prevalence of ACS across deciles of predicted risk**

|  | **Deciles of predicted risk of ≥50% ACS** | | | | | | | | | |
| --- | --- | --- | --- | --- | --- | --- | --- | --- | --- | --- |
|  | ***Derivation: LLS US patients (after internal validation)*** | | | | | | | | | |
| **Predicted prevalence based on DACS-LEAD (%)** | 1.1 | 1.9 | 2.5 | 3.3 | 4.0 | 4.9 | 6.1 | 7.5 | 9.7 | 15.1 |
| **Number of patients with ≥50% ACS** | 54 | 149 | 273 | 370 | 466 | 588 | 740 | 932 | 1133 | 1649 |
| **Observed prevalence (%)** | 0.5 | 1.4 | 2.3 | 3.3 | 4.2 | 5.2 | 6.6 | 8.4 | 10.3 | 14.3 |
|  | ***Validation: LLS UK patients (after recalibration)*** | | | | | | | | | |
| **Predicted prevalence based on DACS-LEAD (%)** | 1.9 | 3.4 | 4.5 | 5.7 | 7.0 | 8.4 | 10.1 | 12.6 | 15.4 | 21.4 |
| **Number of patients with ≥50% ACS** | 7 | 11 | 19 | 30 | 38 | 51 | 59 | 82 | 78 | 116 |
| **Observed prevalence (%)** | 1.3 | 2.1 | 3.7 | 5.2 | 7.0 | 9.4 | 11.0 | 15.2 | 15.4 | 20.1 |
|  | ***Validation: SMART (after recalibration)*** | | | | | | | | | |
| **Predicted prevalence based on DACS-LEAD (%)** | 2.8 | 6.1 | 9.4 | 12.0 | 14.3 | 16.6 | 19.6 | 22.9 | 27.2 | 35.9 |
| **Number of patients with ≥50% ACS** | 4 | 8 | 21 | 23 | 24 | 26 | 28 | 36 | 39 | 49 |
| **Observed prevalence (%)** | 2.8 | 5.1 | 13.0 | 15.0 | 16.3 | 17.7 | 17.1 | 22.9 | 25.3 | 31.8 |
| ACS, asymptomatic carotid stenosis; LLS, Life Line Screening; PACAS-LEAD score, Prevalence of Asymptomatic Carotid Artery Stenosis in patients with Lower Extremity Arterial Disease score; SMART, Second Manifestations of ARTerial diseases. | | | | | | | | | | |

|  | **Deciles of predicted risk of ≥70% ACS** | | | | | | | | | |
| --- | --- | --- | --- | --- | --- | --- | --- | --- | --- | --- |
|  | ***Derivation: LLS US patients (after internal validation)*** | | | | | | | | | |
| **Predicted prevalence based on DACS-LEAD (%)** | 0.5 | 0.8 | 1.0 | 1.3 | 1.6 | 2.0 | 2.5 | 3.2 | 4.4 | 7.4 |
| **Number of patients with ≥70% ACS** | 23 | 53 | 110 | 124 | 206 | 271 | 289 | 381 | 544 | 800 |
| **Observed prevalence (%)** | 0.2 | 0.5 | 1.0 | 1.2 | 1.7 | 2.4 | 2.6 | 3.5 | 4.7 | 7.1 |
|  | ***Validation: LLS UK patients (after recalibration)*** | | | | | | | | | |
| **Predicted prevalence based on DACS-LEAD (%)** | 0.9 | 1.6 | 2.1 | 2.8 | 3.5 | 4.2 | 5.3 | 6.6 | 8.4 | 12.7 |
| **Number of patients with ≥70% ACS** | 2 | 2 | 11 | 14 | 15 | 28 | 35 | 45 | 45 | 64 |
| **Observed prevalence (%)** | 0.4 | 0.4 | 1.8 | 2.6 | 2.8 | 5.2 | 6.5 | 8.4 | 8.6 | 11.4 |
|  | ***Validation: SMART (after recalibration)*** | | | | | | | | | |
| **Predicted prevalence based on DACS-LEAD (%)** | 2.2 | 4.6 | 6.6 | 8.5 | 10.2 | 12.2 | 14.5 | 16.7 | 19.9 | 27.1 |
| **Number of patients with ≥70% ACS** | 2 | 5 | 14 | 14 | 16 | 24 | 22 | 31 | 22 | 41 |
| **Observed prevalence (%)** | 1.4 | 3.1 | 9.5 | 9.5 | 11.9 | 13.2 | 13.9 | 20.3 | 17.3 | 22.7 |
| ACS, asymptomatic carotid stenosis; LLS, Life Line Screening; DACS-LEAD score, Prevalence of Asymptomatic Carotid Artery Stenosis in patients with Lower Extremity Arterial Disease score; SMART, Second Manifestations of ARTerial diseases. | | | | | | | | | | |

**Appendix Figure 1. Flowchart**

Total number of studies included (n = 0)

Records identified through database searching:

*PubMed: n = 324*

*OVID EMBASE: n = 4758*

**Included**

Records after duplicates removed (n = 175)

Records screened
(n = 4907)

Records excluded based on title/abstract (n = 4973)

Full-text articles assessed for eligibility
(n = 43)

**Identification**

**Eligibility**

**Screening**

Full-text articles excluded, with reasons (n = 43)

**Appendix Figure 2. Distribution of sum scores in the derivation cohort**


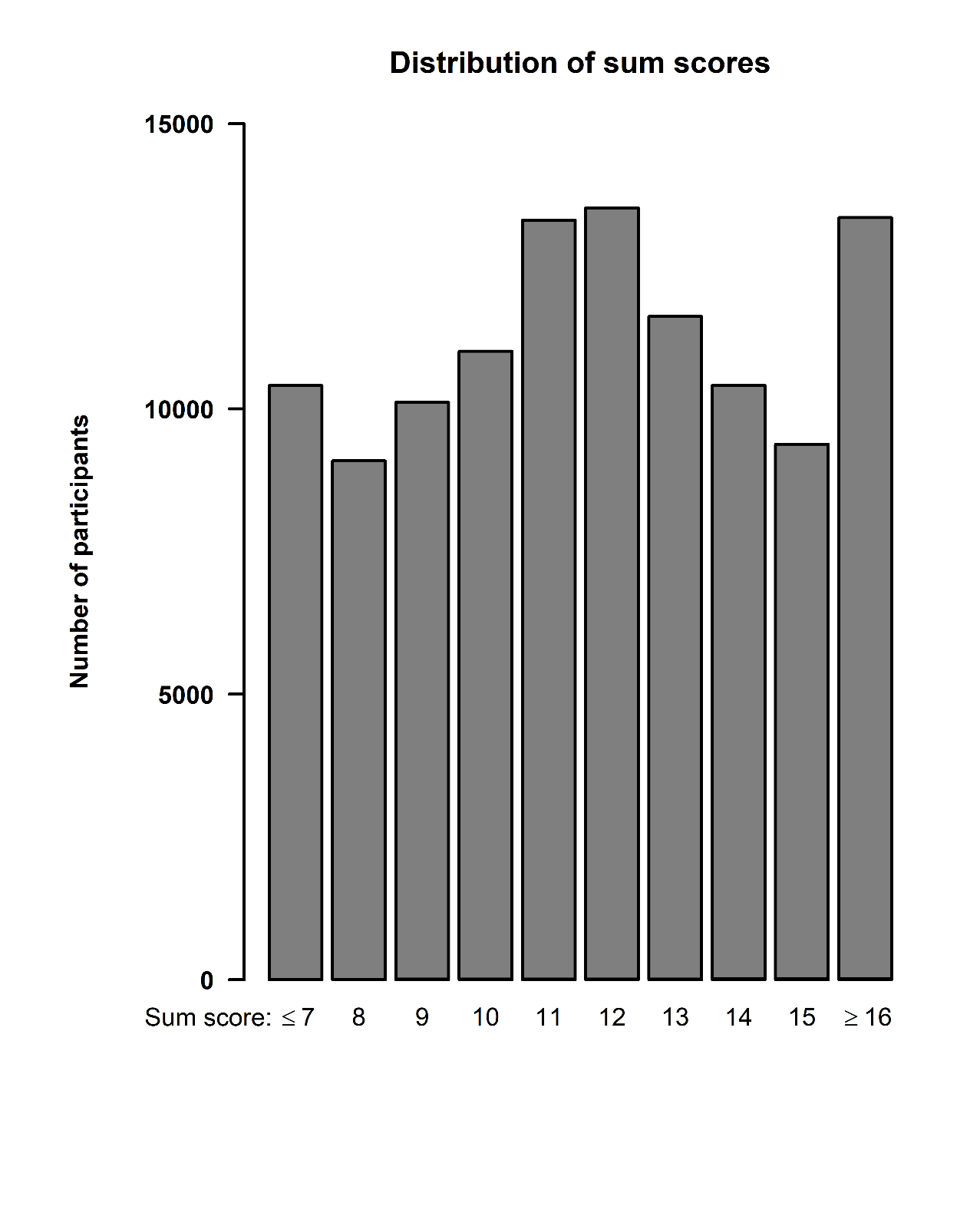


**Appendix Figure 3. Calibration plots of DACS-LEAD in validation cohort (before recalibration)**

Calibration plots showing the predicted risk against the observed risk of ≥50% (*left column*) and ≥70% ACS (*right column*) across deciles of predicted risk in the validation cohorts before recalibration. The boxes represent the mean predicted risk for each decile and the vertical lines represent the 95% confidence intervals. The dotted diagonal line indicates perfect calibration. Boxes above the diagonal line indicate underestimation of risk and below the diagonal line overestimation of risk.

# References

1. Ingui BJ, Rogers MA. Searching for clinical prediction rules in MEDLINE. *J Am Med Inform Assoc* 2001;**8**(4): 391-397.

2. Avci A, Fidan S, Tabakci MM, Toprak C, Alizade E, Acar E, et al. Association between the gensini score and carotid artery stenosis. *Korean Circ J* 2016;**46**(5): 639-645.

3. Carolei A, Marini C, Nencini P, Gandolfo C, Motto C, Zanette E, et al. Prevalence and outcome of symptomatic carotid lesions in young adults. *BMJ* 1995;**310**(6991): 1363-1366.

4. de Weerd M, Greving JP, Hedblad B, Lorenz MW, Mathiesen EB, O'Leary DH, et al. Prediction of asymptomatic carotid artery stenosis in the general population: identification of high-risk groups. *Stroke* 2014;**45**(8): 2366-2371.

5. Greco G, Egorova NN, Moskowitz AJ, Gelijns AC, Kent KC, Manganaro AJ, et al. A model for predicting the risk of carotid artery disease. *Ann Surg* 2013;**257**(6): 1168-1173.

6. Jacobowitz GR, Rockman CB, Gagne PJ, Adelman MA, Lamparello PJ, Landis R, et al. A model for predicting occult carotid artery stenosis: screening is justified in a selected population. *J Vasc Surg* 2003;**38**(4): 705-709.

7. Lacroix P, Aboyans V, Criqui MH, Bertin F, Bouhamed T, Archambeaud F, et al. Type-2 diabetes and carotid stenosis: A proposal for a screening strategy in asymptomatic patients. *Vasc Med* 2006;**11**(2): 93-99.

8. Qureshi AI, Janardhan V, Bennett SE, Luft AR, Hopkins LN, Guterman LR. Who should be screened for asymptomatic carotid artery stenosis? Experience from the Western New York Stroke Screening Program. *J Neuroimaging* 2001;**11**(2): 105-111.

9. Suri MFK, Ezzeddine MA, Lakshminarayan K, Divani AA, Qureshi AI. Validation of two different grading schemes to identify patients with asymptomatic carotid artery stenosis in general population. *J Neuroimaging* 2008;**18**(2): 142-147.

10. Yan Y, Gao S, Yang H, Qin S, Li F, Zhang G, et al. ECAS score: a web-based risk model to predict moderate and severe extracranial carotid artery stenosis. *Neurol Res* 2018;**40**(4): 249-257.

11. Abd Allah F, Baligh E, Ibrahim M. Clinical Relevance of Carotid Atherosclerosis among Egyptians: A 5-Year Retrospective Analysis of 4,733 Subjects. *Neuroepidemiology* 2010;**35**(4): 275-279.

12. Admani AK, Mangion DM, Naik DR. Extracranial carotid artery stenosis: Prevalence and associated risk factors in elderly stroke patients. *Atherosclerosis* 1991;**86**(1): 31-37.

13. Rockman CB, Hoang H, Guo Y, Maldonado TS, Jacobowitz GR, Talishinskiy T, et al. The prevalence of carotid artery stenosis varies significantly by race. *J Vasc Surg* 2013;**57**(2): 327-337.

14. Rockman CB, Jacobowitz GR, Gagne PJ, Adelman MA, Lamparello PJ, Landis R, et al. Focused screening for occult carotid artery disease: patients with known heart disease are at high risk. *J Vasc Surg* 2004;**39**(1): 44-51.

15. Alexandrova NA, Gibson WC, Norris JW, Maggisano R. Carotid artery stenosis in peripheral vascular disease. *J Vasc Surg* 1996;**23**(4): 645-649.

16. Goessens BMB, Visseren FLJ, Algra A, Banga JD, Van Der Graaf Y. Screening for asymptomatic cardiovascular disease with noninvasive imaging in patients at high-risk and low-risk according to the European Guidelines on Cardiovascular Disease Prevention: The SMART study. *J Vasc Surg* 2006;**43**(3): 525-532.

17. Mostaza JM, Gonzalez-Juanatey JR, Castillo J, Lahoz C, Fernandez-Villaverde JM, Maestro-Saavedra FJ. Prevalence of carotid stenosis and silent myocardial ischemia in asymptomatic subjects with a low ankle-brachial index. *J Vasc Surg* 2009;**49**(1): 104-108.

18. Pilcher JM, Danaher J, Khaw KT. The prevalence of asymptomatic carotid artery disease in patients with peripheral vascular disease. *Clinical Radiology* 2000;**55**(1): 56-61.

19. Qiu J, Zhou Y, Yang X, Zhang Y, Li Z, Yan N, et al. The association between ankle-brachial index and asymptomatic cranial-carotid stenosis: a population-based, cross-sectional study of 5440 Han Chinese. *Eur J Neurol* 2016;**23**(4): 757-762.

20. Salonen R, Seppanen K, Rauramaa R, Salonen JT. Prevalence of carotid atherosclerosis and serum cholesterol levels in eastern Finland. *Arteriosclerosis* 1988;**8**(6): 788-792.

21. Cheng SWK, Wu LLH, Ting ACW, Lau H, Wong J. Screening for asymptomatic carotid stenosis in patients with peripheral vascular disease: A prospective study and risk factor analysis. *Cardiovasc Surg* 1999;**7**(3): 303-309.

22. Cina CS, Safar HA, Maggisano R, Bailey R, Clase CM. Prevalence and progression of internal carotid artery stenosis in patients with peripheral arterial occlusive disease. *J Vasc Surg* 2002;**36**(1): 75-82.

23. Hogberg D, Kragsterman B, Bjorck M, Tjarnstrom J, Wanhainen A. Carotid artery atherosclerosis among 65-year-old Swedish men - A population-based screening study. *Eur J Vasc Endovasc Surg* 2014;**48**(1): 5-10.

24. Willeit J, Kiechl S. Prevalence and risk factors of asymptomatic extracranial carotid artery atherosclerosis: A population-based study. *Arterioscler Thromb* 1993;**13**(5): 661-668.

25. Willeit J, Kiechl S, Oberhollenzer F, Rungger G, Egger G, Bonora E, et al. Distinct risk profiles of early and advanced atherosclerosis: Prospective results from the Bruneck study. *Arterioscler Thromb Vasc Biol* 2000;**20**(2): 529-537.

26. Woo SY, Joh JH, Han SA, Park HC. Prevalence and risk factors for atherosclerotic carotid stenosis and plaque a population-based screening study. *Medicine (United States)* 2017;**96**(4): e5999.

27. Yun WS, Rho YN, Park UJ, Lee KB, Kim DI, Kim YW. Prevalence of asymptomatic critical carotid artery stenosis in Korean patients with chronic atherosclerotic lower extremity ischemia: is a screening carotid duplex ultrasonography worthwhile? *J Korean Med Sci* 2010;**25**(8): 1167-1170.

28. Derdeyn CP, Powers WJ. Cost-effectiveness of screening for asymptomatic carotid atherosclerotic disease. *Stroke* 1996;**27**(11): 1944-1950.

29. Derdeyn CP, Powers WJ, Moran CJ, DeWitte ITC, Allen BT. Role of Doppler US in screening for carotid atherosclerotic disease. *Radiology* 1995;**197**(3): 635-643.

30. Jahromi AS, Clase CM, Maggisano R, Bailey R, Safar HA, Cina CS. Progression of internal carotid artery stenosis in patients with peripheral arterial occlusive disease. *J Vasc Surg* 2009;**50**(2): 292-298.

31. Hill AB. Should patients be screened for asymptomatic carotid artery stenosis? *Can J Surg* 1998;**41**(3): 208-213.

32. Jonas DE, Feltner C, Amick HR, Sheridan S, Zheng ZJ, Watford DJ, et al. Screening for asymptomatic carotid artery stenosis: A systematic review and meta-analysis for the U.S. Preventive Services Task Force. *Ann Intern Med* 2014;**161**(5): 336-346.

33. LeFevre ML. Screening for asymptomatic carotid artery stenosis: U.S. Preventive Services Task Force recommendation statement. *Ann Intern Med* 2014;**161**(5): 356-362.

34. Simons PC, van der Graaf Y, Banga JD, Eikelboom BC, Algra A. [The screening for asymptomatic vascular disease and risk factors in high risk patients: current practice]. *Ned Tijdschr Geneeskd* 1998;**142**(19): 1096-1099.

35. Hwang CS, Liao KM, Tegeler CH. A multiple regression model of combined duplex criteria for detecting threshold carotid stenosis and predicting the exact degree of carotid stenosis. *J Neuroimaging* 2003;**13**(4): 324-329.

36. Hwang CS, Shau WY, Tegeler CH. Doppler velocity criteria based on receiver operating characteristic analysis for the detection of threshold carotid stenoses. *J Neuroimaging* 2002;**12**(2): 124-130.

37. Pedro LM, Sanches JM, Seabra J, Suri JS, Fernandes EFJ. Asymptomatic carotid disease - A new tool for assessing neurological risk. *Echocardiography* 2014;**31**(3): 353-361.

38. Goessens BMB, Visseren FLJ, Kappelle LJ, Algra A, Van Der Graaf Y. Asymptomatic carotid artery stenosis and the risk of new vascular events in patients with manifest arterial disease: The SMART study. *Stroke* 2007;**38**(5): 1470-1475.

39. Kakkos SK, Griffin MB, Nicolaides AN, Kyriacou E, Sabetai MM, Tegos T, et al. The size of juxtaluminal hypoechoic area in ultrasound images of asymptomatic carotid plaques predicts the occurrence of stroke. *J Vasc Surg* 2013;**57**(3): 609-618.

40. Kakkos SK, Nicolaides AN, Charalambous I, Thomas D, Giannopoulos A, Naylor AR, et al. Predictors and clinical significance of progression or regression of asymptomatic carotid stenosis. *J Vasc Surg* 2014;**59**(4): 956-967.

41. Kakkos SK, Nicolaides AN, Griffin M, Sabetai M, Dhanjil S, Thomas DJ, et al. Factors associated with mortality in patients with asymptomatic carotid stenosis: Results from the ACSRS study. *Int Angiol* 2005;**24**(3): 221-230.

42. Kakkos SK, Nicolaides AN, Kyriacou E, Daskalopoulou SS, Sabetai MM, Pattichis CS, et al. Computerized texture analysis of carotid plaque ultrasonic images can identify unstable plaques associated with ipsilateral neurological symptoms. *Angiology* 2011;**62**(4): 317-328.

43. Kakkos SK, Sabetai M, Tegos T, Stevens J, Thomas D, Griffin M, et al. Silent embolic infarcts on computed tomography brain scans and risk of ipsilateral hemispheric events in patients with asymptomatic internal carotid artery stenosis. *J Vasc Surg* 2009;**49**(4): 902-909.

44. Smolen HJ, Cohen DJ, Samsa GP, Toole JF, Klein RW, Furiak NM, et al. Development, validation, and application of a microsimulation model to predict stroke and mortality in medically managed asymptomatic patients with significant carotid artery stenosis. *Value Health* 2007;**10**(6): 489-497.

1. https://www.nice.org.uk/guidance/ng50/documents/search-strategies [↑](#footnote-ref-1)
